# Efficient isolation of monocytes from PBMCs utilizing a newly developed material

**DOI:** 10.1186/2051-1426-3-S2-P212

**Published:** 2015-11-04

**Authors:** Xue Zhang, Takayuki Miyamoto, Yoshiko Miura

**Affiliations:** 1 Medical Device Development Laboratories, Kaneka Corp., Takasago, Japan; 2 University of Kyushu, Japan, Fukuoka, Japan

## Background

Recent years immunotherapy has been used to treat various diseases especially cancer. The dendritic cell therapy has been one of the most widely practiced therapies in clinics and usually monocytes are isolated as the source of dendritic cells. However, although dendritic cell therapy shows its effect in cancer treatment, a big problem is one cannot guarantee an enough number of cells for treatment. This is mainly due to the inefficiency of monocyte isolation.

## Methods

Up to now, various methods for selectively isolating monocytes have been developed to trap as many monocytes as possible. For example, a method using a density-gradient centrifugation and a method using magnetic beads with antibodies affinitive to cells have been promoted for several years. However, these methods show rare possibilities to instead the plastic adherence which is the most commonly used method for monocyte isolation. The first reason is proposed to be the complicated operating process. The second reason might be their low cost performance.

## Results

Here we present a new material that gives a high affinity to monocytes but other PBMCs. Plastic dishes or glass dishes coated with this material were used to attract monocytes using a protocol exactly the same with the traditional plastic adherence protocol. The differentiation of monocytes to dendritic cells were carried out by adding GM-CSF and IL-4 to the culture medium and the maturation of those dendritic cells was proceeded by addition of TNF alpha and PGE2.

## Conclusions

As the results indicated, we gained more adhered monocytes comparing with the traditional plastic adherence. Therefore, we believe that this material will be widely used in monocyte isolation and hence contribute to dendritic cell therapy.

